# Telomere Length in Neonatal Dairy Calves in Relation to Lifetime Parameters †

**DOI:** 10.3390/ani15010109

**Published:** 2025-01-06

**Authors:** Manon Dewulf, Luc Duchateau, Maya Meesters, Dries S. Martens, Tim S. Nawrot, Mieke Van Eetvelde, Geert Opsomer

**Affiliations:** 1 Department of Internal Medicine, Reproduction and Population Medicine, Faculty of Veterinary Medicine, Ghent University, Salisburylaan 133, 9820 Merelbeke, Belgiumgeert.opsomer@ugent.be (G.O.); 2 Biometrics Research Group, Department of Veterinary and Biosciences, Faculty of Veterinary Medicine, Ghent University, Salisburylaan 133, 9820 Merelbeke, Belgium; luc.duchateau@ugent.be; 3 Centre for Environmental Sciences, Hasselt University, Agoralaan Gebouw D, 3590 Diepenbeek, Belgium; dries.martens@uhasselt.be (D.S.M.);; 4 Research Unit Environment and Health, Department of Public Health & Primary Care, Leuven University, 3000 Leuven, Belgium

**Keywords:** telomeres, dairy cows, longevity, production, reproduction

## Abstract

Telomere length (TL) has gained attention as a biomarker for longevity and productivity in dairy cattle. This study explored the association between neonatal TL in Holstein calves and lifetime parameters (lifespan, milk production, and reproduction). Blood samples were collected from 210 calves (≤10d old) across four dairy farms in Flanders, Belgium. Telomere length was measured using qPCR and analyzed as a continuous variable and across three groups: the 10% shortest, the 10% longest, and the remaining 80%. Survival analyses showed no association between TL and lifespan (*p* = 0.1) or TL groups (*p* = 0.8). Similarly, TL showed no significant association with production traits. However, categorical analyses revealed that calves with the longest TL had lower lifetime fat (*p* = 0.01) and protein yields (*p* = 0.01) than those with the shortest TL. Reproductive analyses showed cows in the long TL group required fewer inseminations per lactation (*p* = 0.02) and exhibited longer calving intervals (*p* = 0.05). These findings suggest that while neonatal TL may not predict productive lifespan, it may provide insight into reproductive efficiency. Future studies should prioritize longitudinal assessments of TL dynamics to better understand their interactions with management practices and application in herd improvement.

## Simple Summary

Dairy cows are essential for milk production, but their productive lifespan—how long they stay healthy and productive—has not increased in recent years. This has led to concerns about animal welfare, environmental sustainability, and farm profitability. Scientists are searching for ways to predict which calves will have longer, healthier lives. One idea is to measure telomeres: tiny structures at the ends of chromosomes that shorten as animals age. In this study, we measured the telomere length of newborn dairy calves and investigated if it could predict their lifespan, milk production, or reproductive performance. We found that telomere length did not predict how long the cows lived or how much milk they produced. However, calves with the longest telomeres were less efficient at producing milk fat and protein. Interestingly, these calves also required fewer inseminations and had slightly longer intervals between calvings, suggesting a possible link to reproductive performance. While telomere length alone may not be a reliable predictor of lifespan or productivity, it could help us better understand the biology of aging and reproduction in dairy cows. Future research could provide more insights into how this information could improve animal welfare and farming efficiency.

## 1. Introduction

Although the natural life expectancy of dairy cattle is approximately 20 years, the productive lifespan—defined as the period from the animals’ first calving until removal from the herd—typically spans between 2.5 and 4 years in modern dairy industries [1]. The many years of increased focus on milk production in modern dairy cow breeding have been associated with a decline in the length of a cow’s productive life. This emphasis on production has resulted in multiple challenges, including increased health problems, reduced fertility, and compromised animal welfare [2].

Cow lifespan—defined as the period from the animals’ birth until removal from the herd—has attracted growing attention as it contributes to the economic, environmental, and social sustainability of the dairy industry [3]. Longer-living cows offer numerous advantages: they mitigate the expenses and diminish the environmental footprint of dairy operations by reducing the need for rearing replacement heifers and enhancing farm profitability by increasing the number of lactations per cow [2]. A decreased lifespan not only impacts economic and environmental factors but also indicates suboptimal animal welfare, particularly when cows are culled due to health or reproductive issues [1].

Telomeres, repetitive DNA sequences (TTAGGG) interacting with proteins that form a cap at the ends of eukaryotic linear chromosomes, undergo shortening with each cell division [4,5]. Critically short telomeres trigger a DNA damage response, leading to replicative senescence or apoptosis [6]. Telomere length (TL) has emerged as a promising health and longevity biomarker in various fields, such as biomedicine, epidemiology, ecology, and biology [7]. Telomere length is linked to health and survival outcomes across multiple species, with studies in mice showing a survival advantage with increased TL [8]. Recently, TL has also gained attention in high-yielding dairy cattle as a potential marker for longevity [9], productive lifespan [10], and welfare [11].

A significant challenge in enhancing the productive lifespan of dairy cows is the fact that herd managers generally have to rely on metrics that only become available after the first lactation when deciding whether to cull or keep an animal [2]. Identifying indicators of long-term productivity at an early age would allow for better predictions of which cows will have a longer productive lifespan. This would allow farmers to make informed decisions about which offspring to retain, ultimately improving the overall efficiency and productivity of the dairy industry. Therefore, we investigated TL in newborn calves as a potential marker for lifetime parameters. Lifetime parameters in the present study include lifespan, production and reproduction. We hypothesize that TL in neonatal dairy calves is associated with productive lifespan and reproductive performance. To the best of our knowledge, there are currently no longitudinal studies available on TL in dairy cattle, tracking a large cohort of animals from birth to the end of their (productive) life.

## 2. Materials and Methods

### 2.1. Animal Population and Data Collection

The present longitudinal observational study was conducted on 4 dairy farms in Flanders (Belgium) from August 2017 to May 2024. Informed consent was obtained from all participating dairy farmers. The study was conducted according to the guidelines of the Declaration of Helsinki and approved by the Ethical Committee (EC) of the Faculty of Veterinary Medicine (Ghent University, Belgium) under the EC number 2017/87. Furthermore, samples were taken in accordance with the relevant guidelines and regulations, and all authors complied with the ARRIVE guidelines [12]. Informed consent was obtained from all subjects involved in the study.

Farms were selected based on their willingness to collaborate, participation in official monthly milk recording and the availability of necessary data. Herd sizes varied from 100 to 250 lactating cows, with an average 305-day milk yield ranging from approximately 9000 to 11,000 kg. All purebred female Holstein-Friesian (HF) calves born after a gestation period of 265 to 295 days between August 2017 and November 2018 were enrolled in the study. Finally, data from 210 calves were included for further analysis. All included calves were blood sampled ≤10 days of age.

Initial data collection and control involved accurately identifying the calves (Ear Tag ID) and respective herds, and age at blood sampling (in days). All further information (concerning lifespan, productive parameters and reproduction) was extracted from the herd database (Unifarm-Agri, Assen, The Netherlands).

Lifespan parameters included status (present or removed from the herd at the end of the present study), date and concomitant age (in days and months) of eventual removal from the herd, number of lactations (numerical), and eventual culling reason. For all animals, the ‘time to last observation’ was established. For animals that were removed from the herd, this was calculated using the birth date and the date of removal from the herd. For animals still present in the herd at the end of the study period (31 May 2024), the time to last observation in days was determined using the birth date and the end of the study.

Lifetime production parameters included productive lifespan (=day of first calving till day of removal from the herd or end of the study), total amount of milk produced (kg), daily milk yield (=total milk yield divided by productive lifespan (in days)), and total lifetime milk fat and protein production (kg). The average daily production of milk fat and protein (=total lifetime production of fat and protein divided by the number of productive days, in kg) was also calculated from the database. All data on the yield of milk and solids were calculated based on official milk recording data that were based on samplings every 6 weeks.

Reproductive data included the number of parturitions (numerical) and average calving interval (=the average duration between consecutive calvings for a cow over its productive lifetime in days). The average number of inseminations per lactation (=total number of inseminations divided by the number of lactations) was also calculated from the database.

### 2.2. Blood Collection and Laboratory Analyses

Whole-blood samples were taken from the *vena jugularis* within the first 10 days of life in 10 mL Vacutainer^®^ EDTA tubes (Becton Dickinson, Plymouth, UK) using a 20-gauge needle and Venoject^®^ system (Shibuya, Tokyo, Japan). The samples were stored at −30 °C until further analysis.

The samples were analyzed at the Centre for Environmental Sciences, Hasselt University (Hasselt, Belgium), where an interlaboratory comparison of the in-house telomere assay with a US reference lab was performed to standardize the protocol [13]. DNA from whole blood was extracted using the QIAamp DNA Mini Kit (Qiagen Inc., Venlo, The Netherlands). DNA quantity and purity were assessed using a Nanodrop 1000 spectrophotometer (Isogen, Life Science, Utrecht, The Netherlands). The average relative telomere length was measured using a modified singleplex quantitative PCR (qPCR) method adapted from Cawthon [14,15].

DNA integrity was assessed by agarose gel electrophoresis. To ensure a uniform DNA input of 5 ng (accepting range from 4 to 6 ng) for each qPCR reaction, samples were diluted and checked using the Qubit™ dsDNA High Sensitivity Assay Kit (Thermo Fisher Scientific, Bleiswijk, The Netherlands) using the Qubit™ Flex Fluorometer (Thermo Fisher Scientific, Bleiswijk, The Netherlands). All samples were measured as triplicates on a QuantStudio 5 real-time PCR system (Applied Biosystems) in a 384-well format. First, a single copy gene (β globin, HBB) reaction was performed, and this reaction mixture contained a 5 ng DNA template, 1x KAPA SYBR^®^ FAST, Low ROXTM master mix (Kapa Biosystems, Merck) and a 400 nM HBB-forward primer (GAAGGCCCATGGCAAGAAGG) and 400 nM HBB-reverse (CTCACTCAGCGCAGCAAAGG) primer. Cycling conditions were as follows: 1 cycle at 95 °C for 3 min, 40 cycles at 95 °C for 3 s, and 58 °C for 15 s. Second, a telomere-specific reaction was performed, containing a 5 ng DNA template, 1x KAPA SYBR^®^ FAST, Low ROXTM master mix (Kapa Biosystems, Merck), 2 mM DTT, and 100 nM TelG primer (ACACTAAGGTTTGGGTTTGGGTTTGGGTTTGGGTTAGTGT) and 100 nM TelC primer (TGTTAGGTATCCCTATCCCTATCCCTATCCCTATCCCTAACA). Cycling conditions were as follows: 1 cycle at 95 °C for 3 min, 2 cycles at 94 °C for 3 s and 49 °C for 15 s, and 30 cycles at 94 °C for 3 s, 62 °C for 5 s, and 74 °C for 10 s. After each qPCR, a melting curve analysis was performed. On each run, PCR efficiency was evaluated using a standard 6-point serial diluted standard (DNA mixture sample of 10 random DNA samples) curve (efficiencies were 95% for TL and 97% for β-globin with an R^2^ > 0.99 for all standard curves). Five inter-run calibrators (IRCs) were run to account for inter-run variability. After thermal cycling, individual qPCR curves are visually inspected for technical failures and removed in further analyses (*n* = 2 samples). In addition, qPCR triplicate curves that deviate more than 0.3 in Cq value are considered too variable and removed in further calculations (*n* = 20 single curves). The average relative TL was calculated using the qBase+ software (Cellcarta, version 3.2) and expressed as a calibrated normalized relative quantity (CNRQ). The latter is achieved by first calculating the RQ based on the delta-Cq method for telomere (T) and single-copy gene (S) obtained Cq values using target-specific amplification efficiencies. As the choice of a calibrator sample (sample to which subsequent normalization is performed, delta-delta-Cq) strongly influences the error on the final relative quantities (as a result of the measurement error on the calibrator sample), normalization is performed to the arithmetic mean quantification values for all analyzed samples, which results in the NRQ. Samples are measured over different qPCR plates. Therefore, five IRCs are used to calculate an additional correction factor to eliminate run-to-run differences, resulting in the final T/S or ratio (CNRQ). The reaction mixtures used for the telomere run, the single copy-gene run, and the number of PCR cycles used are also described in Meesters et al.’s study (2023) [16].

Mathematical calculation formulas are provided by Hellemans et al. [17]. The method precision is shown by coefficients of variation (CVs) of 0.46%, 0.27% and 6.73% for telomere runs, single-copy gene runs and T/S ratios, respectively. In addition, the intraclass coefficient (ICC) with 95% CI of triplicate measures (T/S ratios) was 0.995 (95% CI 0.994 to 0.997), showing a high repeatability.

Leukocyte TL in relation to a standard reference DNA (T/S ratio) was measured. The T/S ratio (also referred to as ‘relative TL’) is proportional to the mean TL and will be referred to as ‘TL’ throughout the rest of this manuscript.

### 2.3. Statistical Analyses

**Figure 1:**
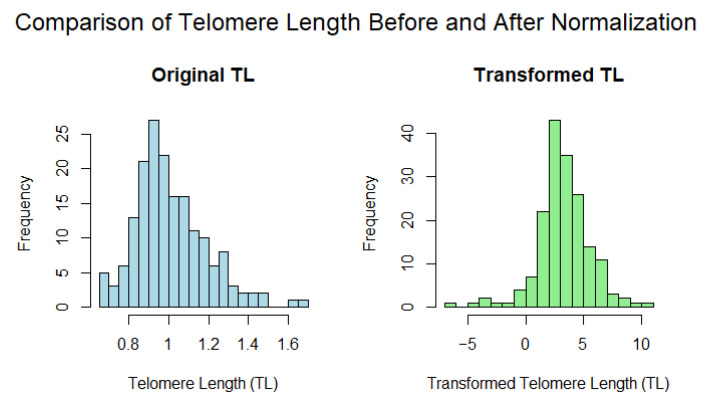
Histograms showing the distribution of telomere length (TL) in calves’ leukocytes before and after normalization. The original TL values (left) were normalized by subtracting the lowest detected TL and multiplying by 10 to create the transformed TL values (right). This transformation was applied to facilitate comparison and standardization across samples (*n* = 210).

Statistical analyses were performed using RStudio 2023.12.1+402. The average leukocyte TL from each calf was normalized by subtracting the lowest detected TL and then multiplying by 10. This transformed TL (Transformed-TL) was set as the unit of interest. The distribution of telomere length in calves before and after normalization can be found in Figure 1. Additionally, the Transformed-TL values were categorized into groups (TL-groups), with the 10% shortest and 10% longest TLs designated as distinct TL groups. Thus, Transformed-TL was analyzed both as a continuous variable and as categorical groups since it is unknown whether the relationship between TL and specific variables is linear.

Lifespan was analyzed using the survival package in R (v3.7-0.; Therneau, 2024) [18]. Status indicates whether the animal was removed (event) or still present in the herd at the end of the study period (right censored). First, Kaplan–Meier plots were generated for visual inspection. Subsequently, Cox proportional hazards frailty models with clustering by herd, i.e., using herd as frailty term, were fitted. Covariates included in the models were season of birth and calf age at sampling (days).

The association between production parameters and Transformed-TL and TL-groups was analyzed using linear mixed-effects models, which were fitted with the lmer() function from the lme4 package in R [19]. The models included herd as a random effect to account for variability between herds, while season of birth and calf age at sampling were included as covariates. Pairwise comparisons between groups were conducted using post hoc tests, and significant differences were indicated using the ‘abc’ system. Groups sharing the same letter are not significantly different (*p* > 0.05), while those with different letters are significantly different (*p* < 0.05).

The average calving interval was analyzed using linear mixed-effects models implemented with the lmer() function in R, with herd included as a random effect and season of birth and calf age at sampling as confounders. The number of parturitions and the average number of inseminations per lactation were treated as repeated measures and analyzed using a Poisson regression model. Pairwise comparisons between groups were conducted using post hoc tests, and significant differences were indicated using the ‘abc’ system.

Model residuals were assessed using a scatterplot of the studentized residuals for homoscedasticity, a linear predictor for linearity, and a Shapiro–Wilk test for normality. The residuals of the models were normally distributed (Shapiro–Wilk *p* > 0.05). The results are expressed as estimates and standard errors. Statistical significance was determined at *p* < 0.05. Data are presented as Mean ± SD unless otherwise stated.

## 3. Results

### 3.1. Descriptives

Blood collection for TL determination was conducted when the neonatal calves were, on average, 4.2 ± 2.21 days old. The TL of 210 included neonatal calves was 1.01 ± 0.173, while the transformed TL was 3.55 ± 1.729. Of these 210 calves, 188 (89%) were inseminated, 178 (84%) conceived, and 174 (83%) reached their first lactation. During the study, 164 (78%) animals were removed from the herd, leaving 46 animals (22%) still present on the farms at the end of the study period (31 May 2024).

### 3.2. Lifespan

The cows were removed after, on average, 1,312 ± 649.9 days or 2.6 ± 1.63 lactations. No association was found between cows removed from the herd and those still present at the end of the study based on Transformed-TL (*p* = 0.5), nor across TL groups (*p* = 0.8). Cows were culled based on five risk factors: disease (*n* = 27), insufficient production (*n* = 8), reproductive failure (*n* = 39), udder health (*n* = 21), and lameness (*n* = 22).

**Figure 2:**
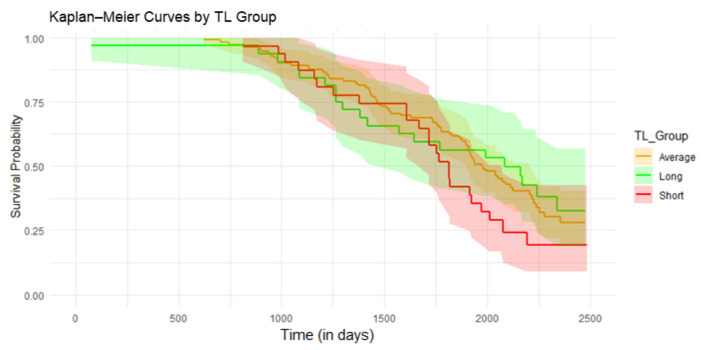
Kaplan–Meier curves showing the association between telomere length (TL) groups and survival. The green curve represents the 10% longest TL, while the red curve represents the 10% shortest TL. The shaded areas around each curve indicate the 95% confidence intervals (*n* = 210).

Survival analyses were conducted on the original 210 animals. Initially, a Kaplan–Meier curve was generated (Figure 2), revealing no survival difference between the TL groups, which was confirmed by the Cox proportional hazards frailty model for Transformed-TL (*p* = 0.1) or across TL groups (*p* = 0.8).

### 3.3. Lifetime Production

Table 1 presents descriptive data for lifetime production and results from the linear models. The analysis of lifetime production was limited to the 128 animals for which lifetime production data were available. When Transformed-TL was analyzed as a continuous variable, no significant associations with production parameters were found. However, when Transformed-TL was categorized, significant negative differences were observed between the 10% shortest and 10% longest TL groups. Specifically, calves in the longest TL group had lower lifetime fat (*p* = 0.01) and protein yields (*p* = 0.01) than those in the shortest TL group.

### 3.4. Reproduction

Table 2 presents descriptive data for reproductive outcomes and results from the linear models. Only the 128 animals, which were removed at the end of the study, were included in the analyses (*n* = 128). When Transformed-TL was analyzed as a continuous variable, associations were found with the average calving interval (Est. ± Std. error = 4.4 ± 0.28 days, *p* < 0.001) and the number of inseminations per lactation (Est. ± Std. error = −0.04 ± 0.01, *p* = 0.005). However, no association was found with the total number of parturitions (*p* = 0.32). Similarly, when Transformed-TL was categorized, differences were observed between the 10% shortest and 10% longest TL groups. Specifically, cows in the long TL group required fewer inseminations per lactation (*p* = 0.007) and had a longer calving interval (*p* < 0.05) compared to those in the short TL group.

## 4. Discussion

In natural populations, longevity denotes the realized lifespan of an animal. However, livestock animals are usually culled at the end of their productive life, so their lifespan measurements must be defined differently than for members of natural populations. Therefore, care must be taken to use the correct nomenclature. Earlier papers have defined “productive lifespan” as the time from first calving to culling, measured in days [1,10]. Telomere length (TL) has emerged as a promising health and longevity biomarker in various species. Consequently, we hypothesized that TL could serve as a predictor of productive lifespan.

The survival analyses show no significant survival difference between the different TL groups, indicating that TL at birth was not significantly associated with cow lifespan in the present study. The lack of a significant association between TL groups and the length of productive lifespan suggests that TL alone may not be a strong predictor of a cow’s risk of being culled. This could be due to several factors.

First, the farmer’s decision to remove animals from the herd introduces a crucial selection bias [18,19]. Ideally, cows would be followed until they reach their natural biological age without intervention, which is not possible in the modern dairy industry. Furthermore, cows are often removed from herds due to multiple reasons such as disease, reproductive failure, as well as udder and claw health. Lastly, lifespan is influenced by numerous genetic and environmental factors beyond TL alone [20].

Second, it may not be static TL at a single time point that predicts longevity but rather the rate of telomere attrition over time. For example, Vera et al. (2012) [21] demonstrate that the rate of TL attrition during an individual’s lifetime, rather than the rate of telomere shortening over time, determines longevity in mice. This concept is further reinforced by a recent study, which concluded that the rate of telomere shortening, rather than the initial telomere length at birth, predicts species’ lifespans [22]. Their results showed that while initial telomere length had no significant correlation with lifespan, telomere shortening rates fit a power law curve with lifespan predictions (R^2^ = 0.934). This finding suggests that species tend to live until their telomeres have shortened to 50–75% of their original length [22]. Longitudinal studies capturing telomere changes might be needed to better predict lifespan in dairy cattle. Similarly, in human medicine, tracking TL from birth to child- and adulthood has been performed [23]. However, there is a clear research gap on TL in relation to lifespan in cattle. To the best of our knowledge, only one other research group has studied TL and its association with productive lifespan and found that heifers at the age of one year with the longest TL had longer productive lifespans [10].

Third, cows, like multiple other species, might possess compensatory mechanisms, such as efficient telomerase activity, which help maintain telomere function despite the presence of shorter telomeres, thus obscuring a direct correlation with longevity [10]. The function and role of telomerase were beyond the scope of the present study but need to be addressed in future research. Furthermore, a short communication by Laubenthal et al. (2016) [24] reports that TL varies across different tissues in dairy cows. However, leukocyte TL has long been recognized as a biomarker of aging in multiple species [25]. Given these established relationships, measuring leukocyte TL in cows offers an easy method, making it particularly relevant in dairy cow research.

Fourth, Vera et al., 2012 [21] highlight that, alongside telomere shortening rates, factors such as body mass and heart rate significantly influence species longevity, a concept supported by numerous studies in bovine medicine identifying various lifespan determinants. For instance, cows born in September and those born from first-parity dams had higher odds of reaching a lifetime milk yield of ≥100,000 kg [26]. Additionally, conformation traits like udder and leg scores seemed to play a crucial role in enhancing the chances of achieving a lifetime milk yield of 100,000 kg and, thus, a longer lifespan [26]. Calving ease and interval are significant for lifespan, as unassisted calving generally leads to longer lifespans [27]. The research of Van Eetvelde et al. (2021) [26] and Hu et al. (2021) [28] clearly indicated that indirect selection criteria play a significant role in the lifespan of dairy cows, possibly even more than the intrinsic genetic potential of the animals. For instance, there are low to moderate genetic correlations between reproductive traits and lifespan, highlighting the importance of these indirect criteria in the selection process [29]. Ultimately, these papers underscore the need for a holistic approach to breeding strategies, integrating both genetic factors and management practices to enhance the lifespan of dairy herds.

In conclusion, multiple factors may explain the lack of association found between TL and lifespan in the present study. Unlike in humans, where TL demonstrates more stability over time, telomere dynamics in cows may exhibit greater variability and faster attrition rates, as evidenced in our longitudinal data showing significant telomere shortening over a relatively short period in dairy cows [30]. This difference likely reflects the rapid cell turnover and accelerated growth characteristic of dairy cows, potentially making a single TL measurement less indicative of long-term outcomes. We conclude that further longitudinal research tracking TL dynamics from birth to adulthood is warranted to establish the true relationship between TL and lifespan in dairy cattle.

The linear models in the present study revealed no significant associations between TL at birth and productivity when analyzed as a continuous variable. However, when TL was categorized, significant negative differences were observed between the 10% shortest and 10% longest TL groups. Specifically, calves in the longest TL group had lower lifetime fat (*p* = 0.01) and protein yields (*p* = 0.01) than those in the shortest TL group. This finding suggests that TL might not be sufficient as a standalone marker for predicting the production of dairy cows.

The relationship between milk production traits and telomere biology appears to be complex. Seeker et al. (2018) [10] examined two genetic groups within their study population that were selected for significant differences in milk yield and found no variation in their mean TL. Our findings align with their research, as no association was detected when TL was analyzed as a continuous variable. These observations imply that TL could potentially be altered through selective breeding without negatively affecting milk yield, although further research is needed to verify this. Environmental factors, management practices, and nutritional influences could play a more direct role in milk production than TL alone [31]. External factors such as stress and overall herd management can have significant effects on milk production [31] and may not be directly related to TL [32]. Additionally, milk production traits are polygenic [32]. The effects of individual genes on milk yield may overshadow any potential influence of TL. Furthermore, in breeding programs, the selection focus is often on traits that directly enhance milk yield and production efficiency. This emphasis on maximizing productivity may inadvertently overshadow or even counteract traits associated with longevity or TL. To address this, future research should incorporate longitudinal studies that assess the impacts of selective breeding on telomere dynamics, longevity, and productivity.

We found a noteworthy relationship between TL and lifetime fat and protein production. This correlation suggests that greater TL may be associated with reduced production of both milk fat and protein. To the best of our knowledge, this is the first study to identify such an association. While these associations are noted, causality cannot be established. The mechanisms behind this association remain unclear, although the link between milk fat and lifespan has already been researched. Kaupe et al. (2007) [33] studied 1291 Holstein cows and found that longevity was phenotypically significantly negatively correlated with milk fat (−0.08). They established the effect of CYP11B1 and DGAT1 on fat and protein content. However, the relationship with lifespan remains to be fully established, but their effects on milk production traits and potential influences on reproductive performance suggest that these genes may play a role in the overall productive lifespan of dairy cows [33]. In contrast, Weigel et al. (1998) [34] found significantly positive genetic correlations between longevity and milk fat (0.46), along with positive correlations with fat and protein (0.56–0.61) as reported by Haile-Mariam and Pryce (2015) [35]. This discrepancy could be explained by potential selection bias among farmers, who may prioritize traits such as high fat and protein content in their breeding decisions. This highlights the importance of indirect selection criteria. Naturally, caution is warranted when interpreting our findings, as the analysis is limited by sample size and the specific production metrics assessed. In conclusion, the significantly negative correlation between TL and average daily milk fat and protein production underscores the complexity of factors influencing dairy cow productivity.

In the present study, we found a relationship between TL and reproductive performance in dairy cows. When TL was assessed as a continuous measure, there was no significant difference in the total number of parturitions. However, our findings indicate a negative association between TL and both the average calving interval and the number of inseminations per lactation. Specifically, with every increase in TL by 0.1, cows required 0.05 fewer inseminations per lactation and had a shorter calving interval of 14.5 days. When TL was analyzed categorically, similar results were obtained: cows with long TLs required significantly fewer inseminations per lactation compared to those with short TLs, with a tendency toward shorter calving intervals in cows with longer TLs. There was no significant difference in the total number of parturitions across TL groups.

These results suggest that cows with longer telomeres may exhibit improved reproductive efficiency, as indicated by the reduced need for inseminations. In human medicine, the link between TL and reproduction is well established. Michaeli et al. (2022) [36] noted that women with longer leukocyte telomeres have a higher likelihood of successful pregnancies at advanced ages (43–48 years) and suggested that TL could serve as a marker of oocyte quality. Longer telomeres may indicate healthier oocytes, thereby improving the chances of successful conception. Additionally, certain genetic factors known to regulate TL are also involved in reproductive functions [37]. Variants in genes such as TERT (telomerase reverse transcriptase) and TERC (telomerase RNA component) can affect both telomere maintenance and reproductive health [37]. Genetic predispositions to shorter telomeres may be linked to reproductive aging and decreased fertility [37]. In bovine medicine, limited research has been performed on the association between TL and reproduction. In other species, reproduction, particularly pregnancy, is associated with TL attrition and accelerated cellular aging, indicating potential ‘costs’ of reproduction. These costs of reproduction on TL have recently been suggested in dairy cows [30]. Overall, these findings underscore the importance of TL as a potential biomarker for reproductive efficiency while highlighting the need for further research in dairy cows.

A major limitation of the present study is the lack of long-term TL tracking. Future research should focus on a larger cohort of cows with extended monitoring to better understand the potential role of telomere dynamics over a cow’s entire lifespan. Additionally, while the study provides valuable insights, the sample size may limit the statistical power to detect smaller effects, particularly when analyzing TL as a categorical variable. A larger sample size could enhance the reliability of results and enable more detailed subgroup analyses, such as exploring potential non-linear relationships. Furthermore, as culling decisions in dairy herds are often influenced by management and economic factors, there is potential for selection bias. Factors unrelated to TL, such as market conditions or individual farmer preferences, may have influenced which cows were removed from the herd, potentially obscuring associations between TL and productive lifespan. Lastly, investigating interactions between telomere length and environmental influences, such as feeding strategies, could provide a more detailed understanding of the factors affecting cow longevity [38]. At the same time, this study did not specifically address the influence of feed on telomere length or lifetime productivity. The selection of farms with comparable production levels and standardized feeding management aimed to minimize variability and focus on the primary research objectives. However, as the study was conducted as a field trial, we acknowledge that this limited our control over all environmental variables.

## 5. Conclusions

In conclusion, the findings of this study emphasize the complex relationship between telomere length (TL) and productive lifespan in dairy cows. While our results indicate that TL at birth does not significantly predict lifespan or productivity, it could inform selection for reproductive efficiency. However, it remains essential to recognize the multifactorial nature of these traits. Factors such as management practices, health status, and environmental influences play critical roles in determining longevity and reproductive efficiency. Furthermore, the dynamics of telomere attrition over time may be more indicative of lifespan than static measurements at a single time point. Results of the present study clearly suggest the necessity for longitudinal studies that capture these changes throughout an animal’s life. As research on TL in dairy cows evolves, integrating insights from both genetics and management strategies will be vital for enhancing the longevity and overall productivity of dairy herds.

## Figures and Tables

**Table 1:** The means and standard errors (SE) across the telomere length (TL) groups. The means were estimated using a linear mixed-effects model fitted with the cell means approach (−1 in the formula) to directly obtain group-specific means. The herd was included as a random effect in the model. Pairwise comparisons between groups were conducted using post hoc tests, and significant differences were indicated using the ‘ab’ system. Groups sharing the same letter are not significantly different (*p* > 0.05), while those with different letters are significantly different (*p* < 0.05) (*n* = 128).

Unit of Interest	Short	Average	Long
Productive lifespan (days)	958 ± 117.0 ^a^	773 ± 42.5 ^a^	584 ± 127.0 ^a^
Lifetime milk yield (kg)	30,255 ± 4550 ^a^	24,144 ± 2280 ^a^	17,277 ± 4610 ^a^
Average milk yield per day (kg)	29.2 ± 2.51 ^a^	30.0 ± 1.99 ^a^	29.4 ± 2.51 ^a^
Lifetime fat yield (kg)	1350 ± 187.0 ^a^	1008 ± 92.5 ^ab^	759 ± 189.0 ^b^
Lifetime protein yield (kg)	1082 ± 156.0 ^a^	831 ± 77.9 ^ab^	613 ± 158.0 ^b^
Average daily milk fat (kg)	4.68 ± 0.168 ^a^	4.21 ± 0.100 ^a^	4.29 ± 0.169 ^a^
Average daily milk protein (kg)	3.52 ± 0.1030 ^a^	3.43 ± 0.0768 ^a^	3.52 ± 0.1040 ^a^

**Table 2:** The means and standard errors (SE) for the reproductive outcomes across telomere length (TL) groups. The means were estimated using a linear mixed-effects model fitted with the cell means approach (−1 in the formula) to directly obtain group-specific means. The herd was included as a random effect in the model. Pairwise comparisons between groups were conducted using post hoc tests, and significant differences were indicated using the ‘abc’ system. Groups sharing the same letter are not significantly different (*p* > 0.05), while those with different letters are significantly different (*p* < 0.05) (*n* = 128).

Unit of Interest	Short	Average	Long
Number of parturitions	3.16 ± 0.193 ^a^	3.31 ± 0.160 ^a^	3.47 ± 0.254 ^a^
Inseminations per lactation	2.64 ± 0.184 ^a^	2.55 ± 0.263 ^b^	2.30 ± 0.134 ^c^
Average calving interval	366 ± 6.8 ^a^	463 ± 62.7 ^b^	383 ± 8.2 ^c^

## Data Availability

The data presented in this study are available upon request from the corresponding author due to privacy and confidentiality agreements with participating farmers and restrictions related to proprietary herd management databases.
